# Coupling conversion of CO/CO_2_ to chemicals through zeolite catalysis

**DOI:** 10.1039/d6sc02673g

**Published:** 2026-05-29

**Authors:** Changcheng Wei, Shaolei Gao, Liang Qi, Zhongmin Liu

**Affiliations:** a National Engineering Research Center of Lower-Carbon Catalysis Technology, Dalian National Laboratory for Clean Energy, Dalian Institute of Chemical Physics, Chinese Academy of Sciences Dalian 116023 Liaoning China qlyanfei920@dicp.ac.cn liuzm@dicp.ac.cn; b University of Chinese Academy of Sciences, Chinese Academy of Sciences Beijing 100049 China

## Abstract

The coupling conversion of CO/CO_2_ (CO_*x*_), sourced from coal, natural gas, biomass, and other carbon sources, with substrates of alcohols, ethers, olefins and alkanes to produce valuable chemicals represents an attractive catalytic route for the direct utilization of CO_*x*_ carbon atoms. The majority of traditional CO_*x*_ conversion processes rely on hydrogenation or carbonylation reactions with metal catalysis. To date, zeolites containing protons in specific atomic scale channels or cages have emerged as one of the most important non-metallic heterogeneous catalysts for the direct coupling of CO_*x*_ with a range of substrates (*e.g.*, alcohols, ethers, olefins and alkanes), yielding products such as acids, esters, ketenes, and aromatics. Different from metal-based catalysis, zeolite-catalyzed CO_*x*_ coupling reactions generally proceed with alkyl cations and acyl cations as key intermediates, the stabilization of which is significantly enhanced within the intrinsic confined zeolitic reaction spaces. Typical processes include dimethyl ether (DME) carbonylation to methyl acetate (MAc), dimethoxy methane (DMM) carbonylation to methyl methoxyacetate (MMAc), olefin carbonylation to branched acids, the reaction of alkanes with CO_*x*_ to aromatics, *etc.* These cases demonstrate the great potential of zeolite in promoting efficient CO_*x*_ coupling. However, despite recent advances in mechanistic studies on DME carbonylation, the fundamental chemistry underlying zeolite-catalyzed CO_*x*_ coupling across widely applied catalytic systems remains insufficiently understood. In this perspective, we summarize decades of research on CO_*x*_ coupling catalysis over zeolites, including reaction mechanisms, catalytic cycles, reaction kinetics and the structure–performance relationships. We also propose future outlooks for achieving a systematic and in-depth understanding of zeolite-catalyzed CO_*x*_ coupling chemistry, optimizing current processes and developing new CO_*x*_ coupling processes.

## Abstract 2

Zeolite-catalyzed CO_*x*_ coupling processes.

## Graphical abstract

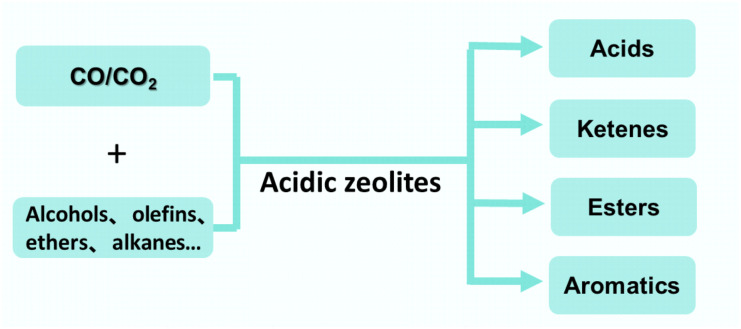

## 1. Introduction

The direct utilization of CO_*x*_, widely generated from carbon resources of coal, natural gas and biomass, for the production of important chemicals is of great significance, and has attracted increasing attention in the past few decades.^1–3^ Traditional processes in this field include Fischer–Tropsch synthesis over a Co- or Fe-based catalyst,^4^ methanol synthesis over a Cu-based catalyst,^5^ methanol carbonylation and olefin hydroformylation processes over Rh- or Ir-based complex catalysts,^6^ methylnitrite carbonylation,^7^ and the reaction of ethylene oxide with CO_2_ to produce ethylene carbonate.^8^ Notably, most of these processes rely on transition-metal or noble-metal-based catalysts.

**Fig. 1:**
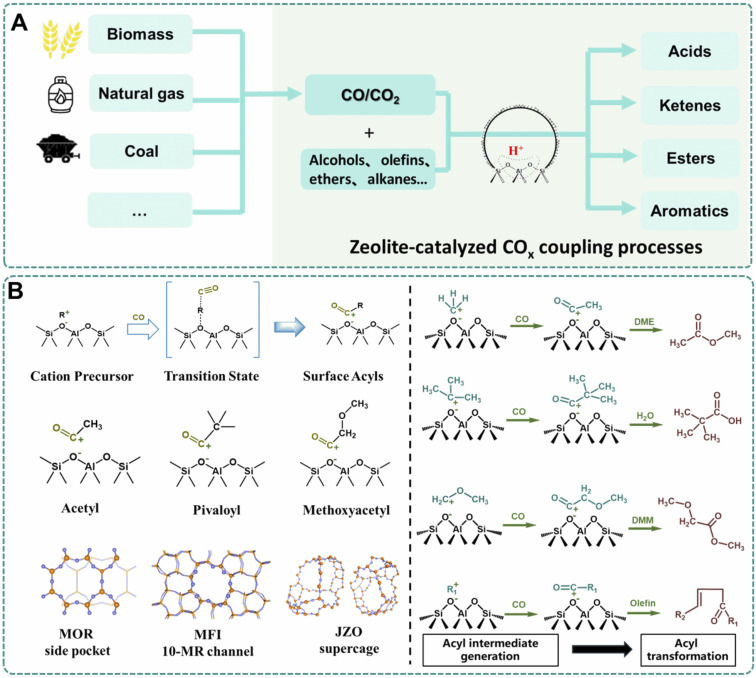
(A) Coupling processes of CO_*x*_ with alcohols, olefins, ethers and alkanes to form acids, ketenes, esters and aromatics. (B) Generation and transformation of acyl species during reported zeolite-catalyzed carbonylation of olefins and ethers.

In early studies, Koch reported that protons or Brønsted acid centers alone can also catalyze the carbonylation of organic substrates.^9–14^ The Koch carbonylation mechanism involved proton-catalyzed formation of carboxylic acids from alcohols, ethers, or olefins through a sequence of electrophilic activation and CO insertion steps. In this process, a proton (H^+^) first attacks a substrate such as an alcohol or olefin to generate a carbocation intermediate. The alkyl cation is then attacked nucleophilically by CO, forming an acyl cation (Fig. 1). Particularly, the Koch reaction favors substrates that can generate stabilized tertiary carbocations, leading predominantly to branched carboxylic acids bearing tertiary carbon centers. In industrial applications, this was primarily utilized for the synthesis of highly branched carboxylic acids such as pivalic acid. Traditionally, the Koch reaction employs strong mineral acids like concentrated sulfuric acid or hydrofluoric acid as catalysts to generate the required carbocation intermediates.^15–17^ However, such conditions posed significant challenges, including severe equipment corrosion, difficult product separation, and environmental hazards.^10,18,19^

Studies by Stepanov and colleagues demonstrated that when *tert*-butanol (*t*-BuOH) or isobutene was co-fed with CO and water over ZSM-5 zeolite, efficient carbonylation occurred, selectively producing tertiary carboxylic acids.^13,14^ The confined environment of zeolite can stabilize reactive carbonyl-containing intermediates while suppressing oligomerization, thus favoring carbonylation over competing pathways. ^13^C solid-state MAS NMR spectroscopy detected the generation of adsorbed acyl species during the reaction, validating the proposed mechanism and the role of zeolite in stabilizing key intermediates.

Besides the carbonylation of alcohols and olefins, it is noteworthy that Brønsted acidic zeolites have recently emerged as the sole metal-free heterogeneous catalysts applicable in continuous-flow reactors for ether carbonylation, driving the rapid advancement of the industrial carbonylation process. Two representative zeolite-based ether carbonylation processes have been reported. One is MOR-catalyzed carbonylation of dimethyl ether (DME) to methyl acetate (MAc), where the active Brønsted acid centers are located in the 8-membered ring (8-MR) side pocket of the zeolite framework.^20–22^ The first commercialization of DME carbonylation was achieved by Dalian Institute of Chemical Physics (DICP) in 2017 (ref. 23 and 24) and the total production capacity of ethanol (*via* MAc hydrogenation) has now reached 4.55 million tons every year. The second example is dimethoxy methane (DMM) carbonylation to methyl methoxyacetate (MMAc) catalyzed by FAU and ZEO-1 zeolites, in which the catalytically active sites are Brønsted acid centers present at channel intersections or within supercages.^25–27^ The process of glycolic acid and methyl glycolate production, based on DMM carbonylation to MMAc followed by MMAc hydrolysis was reported to complete the pilot test in 2022.^27,28^ DMM carbonylation holds great promise for the production of glycolic acid (a monomer for a degradable plastic) or ethylene glycol by MMAc hydration or subsequential hydrogenation and hydration, respectively. In all reported zeolite-catalyzed carbonylation processes, the reactions proceed through acyl cation intermediates stabilized within specific zeolite channels or cages, as listed in Fig. 1. On that basis, the acyl cations undergo further transformation into final products in three distinctive routes: in the presence of water, the acyl cations are hydrolyzed to carboxylic acid; in the presence of ether, they are alkoxylated to form ester; and in the presence of olefin, they are transformed into ketene species.

Besides employing CO for synthesizing oxygenates of acids^13,18^ and esters^25,29^*via* carbonylation in the low temperature range of *ca.* 333–550 K, it has recently been found that zeolite and metal-zeolite catalysts could catalyze coupling conversion of alkanes with CO_*x*_ to produce aromatics^30–33^ at a high temperature of >573 K. In these processes, CO_*x*_ could react with alkyl cations to generate cyclic ketene and/or lactone intermediates, which can be further transformed into aromatics. The formation of cyclic ketenes and/or lactones competes with the hydrogen transfer process, thereby suppressing the generation of light alkanes while promoting aromatics formation. More importantly, carbon atoms from CO_*x*_ can be directly incorporated into the aromatic products *via* cyclic ketene or lactone intermediates, followed by an isomerization process, thereby enabling the efficient valorization of CO_*x*_.

These cases highlight the potential of zeolites in the coupling conversion of CO_*x*_ to produce a series of valuable chemicals, including carboxylic acids, esters, ketenes, and aromatics, attracting intense interest from both academia and industry.^34–36^ In this perspective, we summarize recent advances in CO_*x*_ utilization for chemical production *via* zeolite-based carbonylation catalysis, with a primary focus on reaction mechanism, kinetics, and structure–performance relationship for representative processes including carbonylation of DME and DMM to esters and olefins and alcohols to acids and ketenes, as well as the coupling of alkanes with CO_*x*_ to aromatics *via* carbonylation intermediates. Finally, we provide the main perspectives on the future development of this field toward a systematic and general understanding of zeolite-catalyzed CO_*x*_ coupling reactions.

## 2. Olefin and alcohol carbonylation to carboxylic acids

**Fig. 2:**
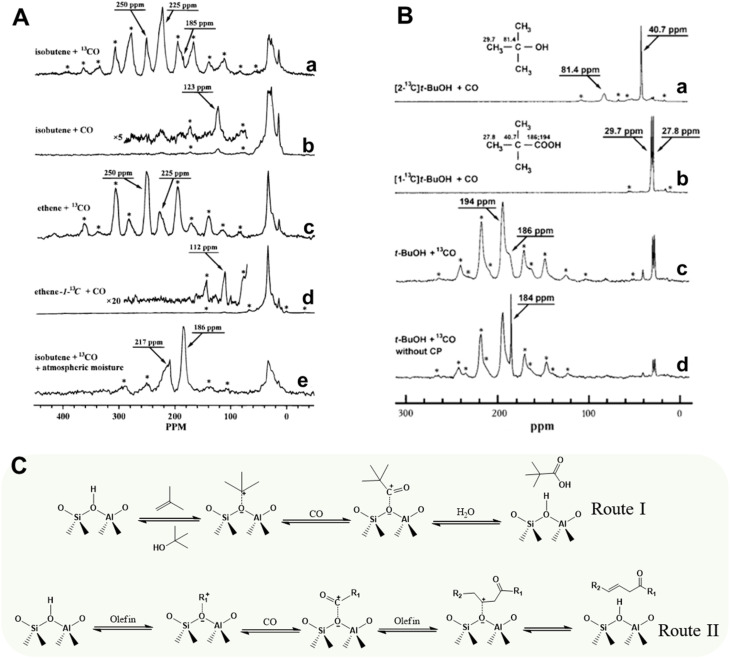
(A) ^13^C CP/MAS NMR spectra of the products formed at 296 K after co-adsorption of olefin and CO on ZSM-5 zeolite: (a) co-adsorption of unlabeled isobutene and ^13^CO; (b) co-adsorption of unlabeled isobutene and unlabeled CO; (c) co-adsorption of unlabeled ethene and ^13^CO; (d) co-adsorption of ethene-1-^13^C and unlabeled CO; (e) after sample (a) was kept for 1 month under an air atmosphere at ambient temperature (225 ppm assigned to the C=O of unsaturated ketones). Reproduced from ref. 11 with permission from American Chemical Society, copyright 1996. (B) ^13^C CP/MAS NMR spectra of the products formed after co-adsorption of *t*-BuOH and CO on ZSM-5 zeolite at 296 K: (a) co-adsorption of *t*-BuOH, ^13^C-labeled in the quaternary carbon atom, and unlabeled CO; (b) co-adsorption of *t*-BuOH, labeled with ^13^C in a methyl group and with unlabeled CO; (c) co-adsorption of ^13^C-labeled CO and unlabeled *t*-BuOH; (d) one pulse excitation ^13^C MAS NMR spectrum with high-power proton decoupling, recorded after co-adsorption of the ^13^CO and unlabeled *t*-BuOH. Reproduced from ref. 13 with permission from Elsevier, copyright 1996. (C) Proposed mechanism of the reaction of olefins and CO in the presence of (Route I)^11,13^ or without the presence of (Route II)^11^ H_2_O.

Carboxylic acid compounds can be produced over H-type zeolites *via* the Koch mechanism, through the reaction of CO with olefins,^11,14^ alkanes^37^ or their derivates.^38,39^ Stepanov and coworkers^13^ first reported that ZSM-5 zeolite catalyzed the carbonylation of alcohols and olefins to carboxylic acids, with the formation of products over the zeolite surface directly observed by ^13^C solid state MAS NMR (Fig. 2A and B). They found that the Koch reaction could occur even at room temperature when *t*-BuOH or isobutene and water were co-fed with CO. The reaction between CO and *tert*-butyl cations gives rise to acyl species, which is favored over isobutene oligomerization owing to the steric confinement effect of zeolites. Further NMR studies using ethylene, isobutene and octene-1 as substrates revealed that tertiary carboxylic acids were invariably formed as the principal products, unless the olefins were too bulky to generate sterically demanding tertiary moieties within the zeolite channels. Moreover, water plays a critical role in determining the product distribution, which enables hydrolysis of acyl cations to the desired carboxylic acid (Route I in Fig. 2C), whereas anhydrous conditions favor ketene formation (Route II in Fig. 2C).^11^

Despite these promising findings, most reported carbonylation processes of olefins, alcohols and alkanes have only been identified by *in situ* spectroscopic investigation, rather than realized as practical and efficient transformations. With the rapid advancement of zeolite and metal-zeolite materials in recent years,^29,40^ the development of efficient olefin carbonylation based on zeolite catalysis is highly expected. To this end, deeper insights into the detailed structure–performance relationships for olefin/alkane carbonylation toward carboxylic acids are essential. Influences of the local environment of Brønsted acid centers within zeolite channels or cages, the nature of incorporated metal species (chemical structure, coordination geometry, *etc.*), and the synergy between metal species and Brønsted protons during the formation and transformation of acyl intermediates, are suggested to be considered in future research.

## 3. Carbonylation reactions mediated with acetyl cations

### 3.1 DME carbonylation to MAc

DME carbonylation represents the first reported ether carbonylation reaction catalyzed by zeolites. In 2006, Iglesia *et al.* first reported that acidic zeolites without metals can catalyze DME carbonylation to MAc with high efficiency.^41^ DME carbonylation over acidic zeolites generally involves three elementary steps:^21^ (1) formation of surface methoxy groups; (2) nucleophilic attack of CO on methoxy groups to generate acetyl intermediates; (3) methoxylation of acetyl intermediates to yield MAc, accompanied by regeneration of the methoxy precursors. DME initially adsorbs onto Brønsted acidic protons of zeolites and subsequently dissociates into surface methoxy and methanol, giving rise to a distinct induction period.^42,43^ During the steady reaction period, methoxylation of the acetyl cations by DME produces MAc and regenerates methoxy groups, with no methanol generated. Among these steps, formation of acetyl intermediates is regarded as the rate-limiting step, supported by kinetic observations that the MAc formation rate is proportional to CO partial pressure but independent of DME partial pressure.^41^

Both the catalytic activity and selectivity for DME carbonylation are sensitive to zeolite topology, and only zeolites with protons located within 8-MR channels (such as FER and MOR^44,45^) are found to be efficient for DME carbonylation.^40,46^ This is attributed to the confinement effect on promoting acetyl cation formation, together with the suppression of side reactions (methanol/DME to hydrocarbons), over protons within the confined 8-MR reaction space.

FER zeolite possesses a two-dimensional microporous framework consisting of 8-MR channels (3.5 × 4.8 Å) along the *b*-axis and 10-MR channels (4.2 × 5.4 Å) along the *c*-axis.^47–49^ Experiments and theoretical calculations have demonstrated that the Brønsted acid sites in the 8-MR channels are also assumed to be the main active sites for catalyzing DME carbonylation.^50–52^ Enrichment of Al atoms in the 8-MR channels of FER zeolite has been proven to effectively enhance catalytic performance. Bae and co-workers reported the synthesis of a highly crystalline FER zeolite enriched in active sites within the 8-MR channels using a seed-assisted method, which exhibited superior catalytic activity and stability.^52^ Several research groups have attempted to manipulate Al siting in 8-MR channels of FER by employing different structure-directing agents (SDAs). Shen *et al.* synthesized FER-type zeolite using dioxane as the SDA, and more than half of the Al atoms were found to be located at T4 sites in the 8-MR pore, which are generally regarded as the active sites for DME carbonylation over FER, and this sample brought about obvious improvement of DME carbonylation activity relative to commercial FER.^48^ Fan *et al.* reported that using morpholine as the template agent could cause more Al siting in 8-MR channels of FER, promoting DME carbonylation by forming more acyl species.^45^ In addition, alleviating the diffusion limitation of micropores by reducing the crystal size can also improve the catalytic performance.^49^ Wu *et al.* fabricated FER nanosheets, which exhibited improved DME conversion, stability, and lifetime in the DME carbonylation reaction.^49^ Although the interconnected 8 × 10-MR pores of FER suppress coke formation, thereby conferring greater stability for DME carbonylation compared to MOR zeolite, FER zeolites have relatively lower catalytic activity in DME carbonylation.^53^

The framework of MOR zeolite consists of 12-MR (6.5 × 7.0 Å) straight channels and flattened 8-MR (2.6 × 5.7 Å) channels along the *c*-axis, interconnected by 8-MR side pockets (3.4 × 4.8 Å) along the *b*-axis.^54^ Owing to the small aperture, most reactants cannot diffuse into the 8-MR channels but instead access active sites *via* the 12-MR straight channels. Subsequently, DME carbonylation proceeds selectively within the 8-MR side pockets that connect the 8-MR and 12-MR channels,^42^ a consequence of the unique spatial confinement necessary for the carbonylation reaction.^22,55^ Despite the selective occurrence of carbonylation within the side pockets of MOR, there exists severe carbon deposition and deactivation over Brønsted acid sites (BASs) located in 12-MR channels, severely compromising catalytic stability.^20^ Therefore, an efficient MOR zeolite-based catalyst for DME carbonylation should feature enrichment of protons in 8-MR side pockets while reducing, passivating, or eliminating those in 12-MR channels.

Great efforts have been dedicated to the direct synthesis of MOR zeolites with tailored morphologies and acid properties,^56^ mainly focusing on two strategies: fabricating hierarchically porous or nanosized MOR crystals to improve the mass transfer of guest molecules,^57–62^ and optimizing the aluminum (Al) distribution to enrich BASs within 8-MR side pockets.^63^ Reviews for MOR synthesis with enhanced DME carbonylation performance have been well summarized in recent publications.^29,40^ Through rational selection of organic templates and optimization of synthesis conditions, the fraction of BAS in 8-MR side pockets can be increased to 54–70.8%.^64–67^ Although such BAS enrichment in 8-MR side pockets brings about enhanced DME carbonylation activity, residual BASs in 12-MR channels still triggers severe side reactions, thus lowering both DME carbonylation selectivity and stability. Therefore, post-treatment processes are still needed to selectively poison or eliminate BASs in the 12-MR channels.

Selective passivation of BASs by alkaline molecule modification^68–71^ and selective removal of framework Al^72^ represent the two dominant post-synthetic strategies for MOR modification. Shen *et al.* reported that DME carbonylation stability over MOR could be significantly improved by pre-adsorption of pyridine.^73^ Other organic alkaline molecules such as alkyimidazolium ions^74^ and tetramethylammonium (TMA^+^) ions^75^ were also selected to be introduced into the channels of MOR *via* ion-exchange. Owing to steric hindrance, these alkaline molecules selectively titrate the acidic protons in 12-MR channels but were inaccessible to the BASs in 8-MR side pockets, accounting for the significantly prolonged catalyst lifetime.

The framework Al of zeolites could be selectively knocked out by multiple post-processing methods. Shen *et al.* reported that framework Al in 8-MR pores of MOR could be protected by Na^+^ ions, while those in 12-MR channels could be selectively removed through high-temperature steam treatment.^72^ Liu's group^76^ proposed a strategy to remove BASs within 12-MR channels of MOR through trimethylchlorosilane (TMCS) treatment. Due to its molecular size limitations, TMCS selectively diffuses into the 12-MR channels of MOR and bonds to the framework Al through a hydrolysis reaction between Si–Cl bonds and acidic protons, thereby passivating the BAS in 12-MR. They further proposed that a low partial pressure SiCl_4_ treatment strategy could promote the migration of framework Al in MOR to T_3_ sites, which are favorable for the DME carbonylation reaction.^77^ Consistently, SiCl_4_ molecules selectively diffuse into the 12-MR channels, and react with framework Al to generate AlCl_3_. The generated AlCl_3_ could migrate through the side pockets into the 8-MR, resulting in about 90% of active sites being located within the 8-MR channels under optimal modification conditions. These strategies effectively increase the ratio of framework Al in 8-MR to that in 12-MR, enhancing the activity, selectivity and stability of DME carbonylation.

**Fig. 3:**
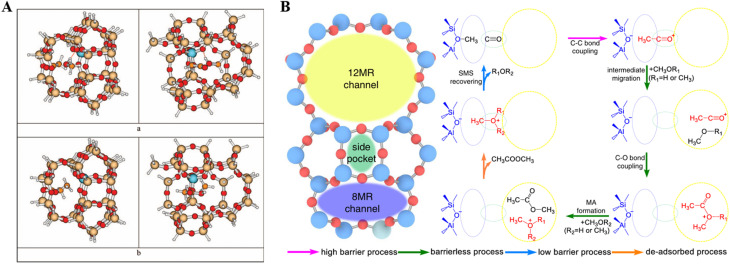
(A) (left) Side views and (right) face views of the optimized structures of the two different transition states obtained for the reaction of methanol with the acylium cation intermediate formed at the T3-O33 position in MOR: (a) reaction products are MAc and a BAS; (b) reaction products are acetic acid and a methoxy group. Reproduced from ref. 22 with permission from American Chemical Society, copyright 2008. (B) Mechanism of DME/methanol carbonylation through the synergistic action of 8-MR channels, side pockets, and 12-MR channels in MOR zeolite.

A clear understanding of the subsequent transformation mechanism of acetyl cations is crucial for optimizing DME carbonylation performance. Corma *et al.*^22^ proposed that methanol or DME attacked acetyl cations to produce MAc with the regeneration of BASs or surface methoxy precursors at the T3-O33 site (Fig. 3). The calculated energy barriers were 144.34 kJ mol^−1^ when attacked by methanol and comparably high if attacked by DME. Zheng *et al.* further found that acetyl cations generated in the 8-MR side pocket could migrate to 12-MR channels, where their conversion by methanol or DME is more favorable.^29,78^ The free energy barriers for acetyl cations migration were 47.9 and 38.8 kJ mol^−1^, respectively, in the presence of DME or methanol, both of which are sufficiently low to enable facile occurrence. Consequently, the reduced steric hindrance of 12-MR channels lowers the energy barriers for the conversion of acetyl cations attacked by methanol or DME to 105.2 kJ mol^−1^ and 115.0 kJ mol^−1^, respectively.

### 3.2 Other carbonylation reactions mediated by acetyl cations

**Fig. 4:**
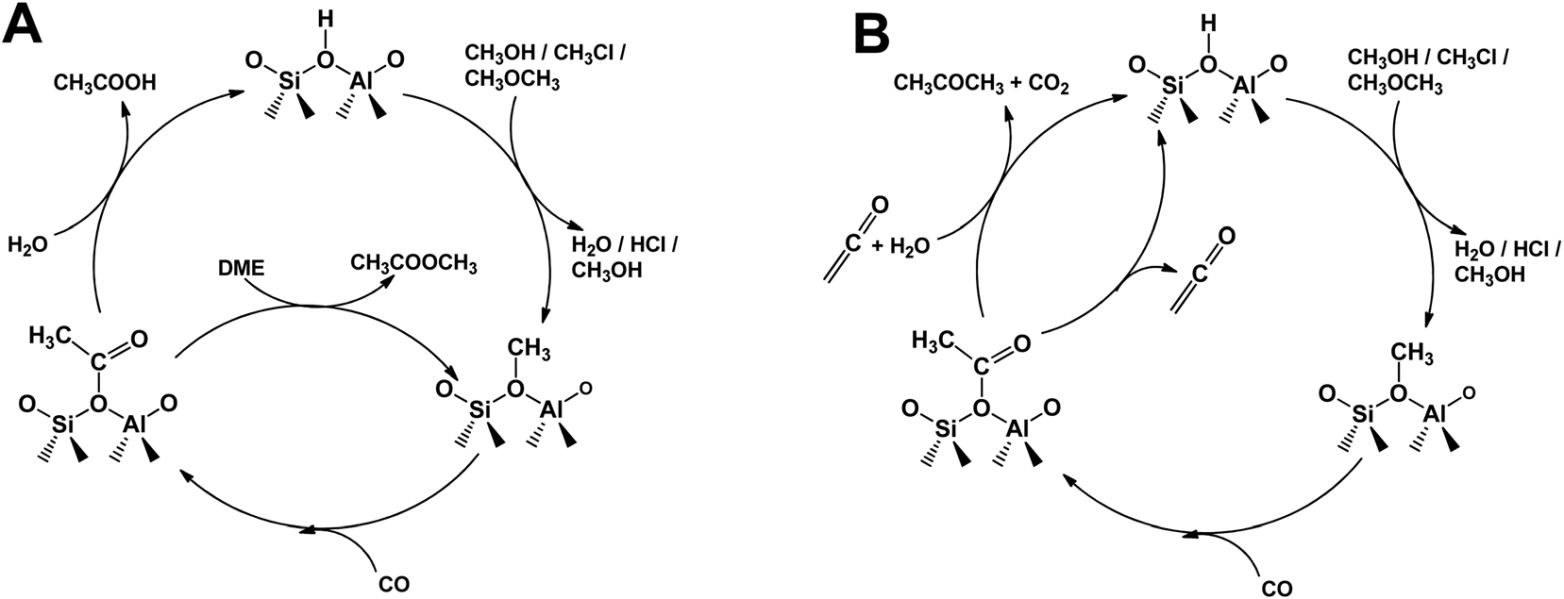
The carbonylation reactions mediated by acetyl cations to form acetic acid/MAc (A) and acetone (B) over acidic zeolites.

According to the aforementioned mechanistic insights into DME carbonylation, efficient DME carbonylation over MOR relies on both the rapid generation of acetyl intermediates and the facile transformation of acetyl species with DME to form MAc products. On that basis, in addition to DME carbonylation to MAc, other carbonylation processes mediated by acetyl cation intermediates have also been reported over MOR zeolite (Fig. 4).

The carbonylation of methanol – one of the most important C1 species – over zeolites was first reported by Fujimoto *et al.* in 1984.^12^ In this process, methanol first generates surface methoxy and water on the catalyst surface, and CO is then inserted into methoxy groups to form acetyl species, which ultimately undergo hydrolysis or esterification to produce acetic acid or MAc. In 1996, researchers from British Petroleum Company reported the methanol carbonylation reaction performance over MOR and Cu/MOR, with a selectivity toward acetyl-containing products (acetic acid + MAc) of above 70%.^79^ Ni *et al.*^39^ also demonstrated that methanol carbonylation could be achieved over MOR and the selectivity to acetic acid could reach up to 95%. They further found that the conversion rate of methanol began to decrease significantly after 15 h, while the conversion rate of methanol over pyridine-adsorbed MOR could be maintained at 100% for at least 145 h.

Surface methoxy groups, which serve as precursors for acetyl group generation, could also be derived from methane or its derivatives, representing a promising route for upgrading methane to oxygenates. In 2007, trace-level conversion of methane with CO to give acetic acid on a solid acid catalyst (sulfated zirconia) was found *via* solid-state NMR.^80^ With copper exchanged MOR zeolites, methoxy species were generated on the oxidation-active copper species during methane carbonylation and subsequently migrated to BASs located in the 8-MR side pockets, where they are converted to acetyl groups in the presence of CO.^81^ Chloromethane, an important derivative of methane that can be obtained from methane *via* halogenation or oxidative halogenation under mild conditions, exhibits high activity and selectivity in carbonylation reactions. A novel one-step route for highly selective preparation of acetic acid from halogenated methane was reported over MOR.^38^ Under optimized conditions, the total selectivity of acetic acid and MAc reached 99.3%, realizing efficient conversion of chloromethane to oxygen-containing compounds. The BASs located within the 8-MR side pockets of MOR are identified as the active site for chloromethane carbonylation. Consistent with that developed for DME carbonylation, the reaction mechanism for chloromethane carbonylation is proposed as follows: chloromethane dissociates and adsorbs to form methoxy groups; CO then inserts into the methoxy groups to generate acetyl species, which subsequently undergo hydrolysis to produce acetic acid (Fig. 4A).

Jensen *et al.*^82^ identified that ketene (CH_2_=C=O), generated from the deprotonation of acetyl, is an important reaction intermediate during DME carbonylation, which is predicted by DFT calculations and further verified experimentally by the observation of doubly deuterated acetic acid (CH_2_DCOOD), when D_2_O was added. Zheng *et al.*^83^ reported that in the 8-MR side pockets of MOR, ketene underwent rapid protonation, forming a stable acylium ion, which is conducive to achieving high reaction activity in MAc and acetic acid formation. However, within the 12-MR channels of MOR, ketene had a relatively longer lifespan, and its conversion within these pores led to carbon deposition, accelerating the deactivation of the reaction. It is worth noting that there exists a dynamic equilibrium between acetyl and ketene over MOR. If acetyl could not react with DME in time to produce MAc and excite the reaction system, this dynamic equilibrium would favor the formation of ketene. Ketene will then undergo polymerization and decarbonylation reactions to generate cyclic oxygen-containing compounds, accounting for the catalyst deactivation.^70^ According to these results, in order to achieve the long lifetime of DME carbonylation, DME conversion should be regulated within a certain range, in which the generated acetyl could react with sufficient DME, preventing the deactivation caused by ketene polymerization and decarbonylation. Moreover, ketene was an important intermediate for the acetone formation during the co-reaction of DME and CO. Zhou *et al.* reported that the high selectivity (73%) of acetone could be achieved over pyridine-modified MOR.^84^ As shown in Fig. 4B, the insertion of CO into surface methoxy species formed acetyl groups, which could be transformed into ketene *via* deprotonation. The generated ketene could further react with acetyl to produce acetone in the presence of H_2_O.

In general, acetyl cations generated from methanol, chloromethane and DME serve as key intermediates or precursors for the synthesis of oxygenated compounds such as carboxylic acids, ketenes and esters. As shown in Fig. 4, high selectivities toward acetic acid, MAc and acetone could be achieved by introducing H_2_O, DME and H_2_O + ketene, respectively, into acetyl cations over zeolites.

## 4. DMM carbonylation to MMAc

Acid catalyzed carbonylation of DMM, a diether molecular that can be regarded as an anhydrous formaldehyde inserted into a DME molecular, offered a mild and efficient pathway to produce MMAc, the precursor to glycolic acid, and glycolic acid and its methyl ester were monomers for producing the biodegradable plastic, polyglycolic acid (PGA). In addition, MMAc could also be directly hydrogenated and hydrolyzed to produce the bulk chemical, ethylene glycol. Therefore, DMM carbonylation is an important process for preparing biodegradable plastic monomers and plays an extremely important role in the chemical industry chain.^27,85^

**Fig. 5:**
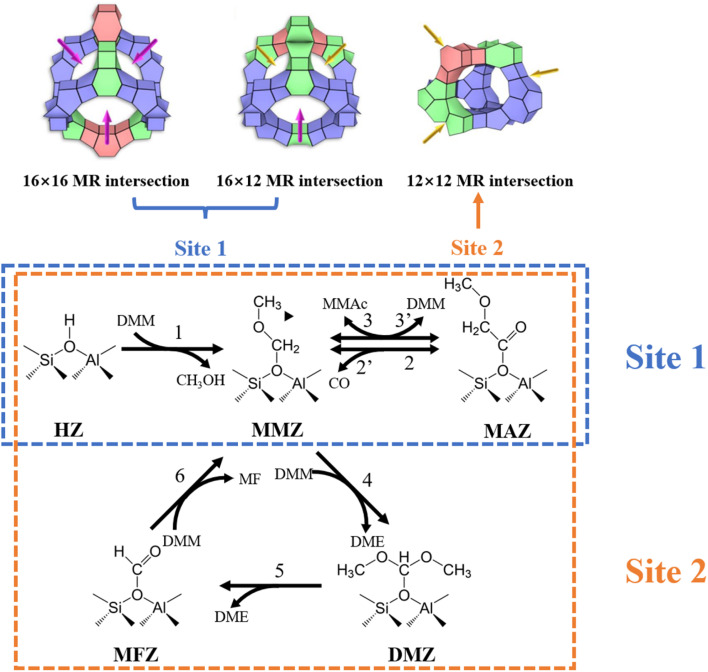
Proposed mechanism for DMM carbonylation and disproportionation over different active sites in ZEO-1. Reproduced from ref. 27 with permission from Elsevier, copyright 2025.

In 2009, Alexis T. Bell *et al.*^25,26,86^ first found that DMM carbonylation can be promoted by zeolites. The possible mechanism of DMM carbonylation is shown in Fig. 5. Firstly, DMM molecules were adsorbed onto the surface of the zeolites through hydrogen bonding; secondly, the interaction between DMM and BASs led to the formation of methoxy methylene species (MMZ) on the catalyst surface and methanol in the gas phase; the nucleophilic attack of CO on MMZ generated the methoxyacetyl intermediate (MAZ). Subsequently, MAZ interacted with another DMM molecule to form the MMAc product, and at the same time, regenerated the MMZ precursor.1
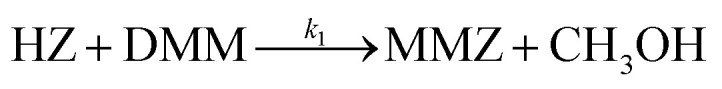
2
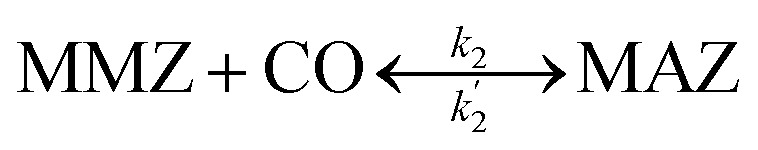
3
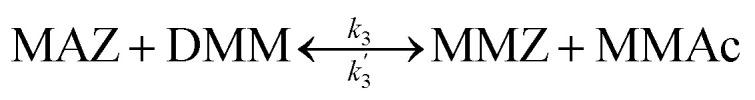
4
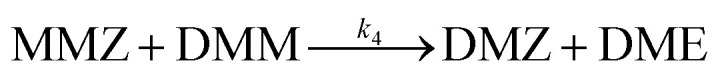
5
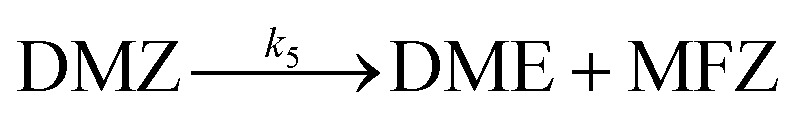
6
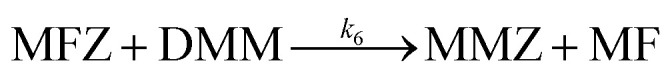
7
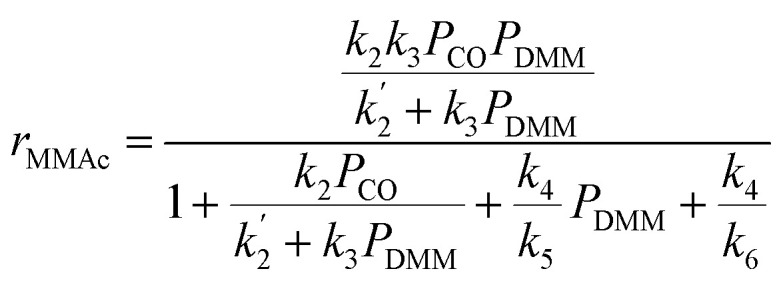
8
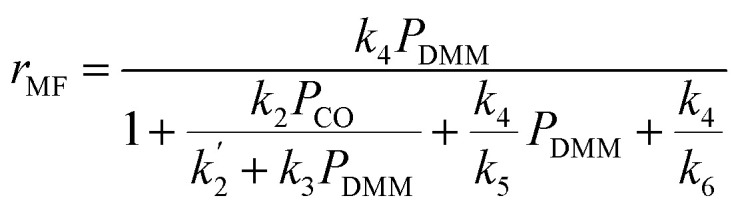


Kinetics experiments^26,86^ showed that the carbonylation and disproportionation rate of DMM was positively correlated with CO partial pressure and DMM partial pressure, respectively. In contrast, carbonylation rate of DMM displayed a zero to negative dependence on DMM partial pressure, while disproportionation rate displaed a negative dependence on CO partial pressure, over MFI and FAU zeolites. Elementary steps (reaction (1)–(6)) and kinetic equations (eqn (7) and (8)) of DMM carbonylation and disproportionation were consistent with experimental results and are shown above. *P*_x_, x = CO or DMM, indicated the partial pressure of reactants. *k*_*x*_ represented the corresponding elementary reaction rate constant. Reaction 2, the formation of the MAZ intermediate was considered as the rate-determining step for DMM carbonylation, while DMM disproportionation was rate-limited by reaction (4), the hydrogen transfer reaction of MMZ.

DMM carbonylation and disproportionation activity were found to be highly relevant to the size of zeolite cages or channels and to Si/Al ratios. Comparative investigation of DMM carbonylation over zeolites such as FAU, BEA, MOR, MFI and FER showed that DMM carbonylation activity and selectivity increase with the increase in channel or cage size, and FAU was tested as the most effective zeolite.^26^ Besides, Yao *et al.*^87^ reported that the Brønsted acid sites in 8-MR side pockets of MOR could be selectively covered by Na^+^ ion exchange, achieving more than 50% DMM conversion and 78 wt% MMAc selectivity, much higher than those of the parent MOR. Xie *et al.*^88^ found that only 10.1–18.3% Al of FAU zeolite contributed to the BAS, and MMAc selectivity showed strong dependences on the strength of BASs. Chen *et al.* found that higher DMM carbonylation activity and selectivity could be achieved over hierarchical HY.^89,90^

The influence from zeolite topology of MFI and FAU^86^ showed that the energy barrier for MAZ intermediate formation on FAU was 68 kJ mol^−1^, while it was 55 and 64 kJ mol^−1^ within MFI sinusoidal and straight channels. In contrast, the methoxylation energy barrier for MAZ intermediates on FAU was 16 kJ mol^−1^, while on MFI, it was 68 kJ mol^−1^. These results demonstrated that, while the activation barriers of MMZ carbonylation to MAZ over MFI and FAU zeolites were comparable, the activation barrier for methoxylation of MAZ was significantly lower for FAU than that for MFI. Energy decomposition analysis revealed that this arose from greater electronic stabilization of the transition state for MAZ in FAU compared to MFI. The zeolite topology also imposed an obvious influence on the DMM disproportionation, the only side reaction of DMM carbonylation. Relative to FAU, the smaller pores of MFI, MOR, BEA, *etc.*, forced the reactants into an orientation that promoted the hydrogen transfer process, the critical step of DMM disproportionation. And therefore, FAU exhibited the highest activity for DMM carbonylation but the lowest activity for DMM disproportionation. The zeolite Si/Al ratio also influenced the rate of MMAc formation over zeolites. As reported, zeolites with lower Al density exhibited higher carbonylation rates,^26^ as the greater spatial separation between Al centers and adsorbed species mitigated repulsive electrostatic interactions. The closer proximity of surface species was expected to increase the activation energy for the cationic transition state involved nucleophilic attack during DMM carbonylation and disproportionation. Therefore, synthesizing zeolites with large reaction space and a high Si/Al ratio was more appropriate in this reaction.

**Fig. 6:**
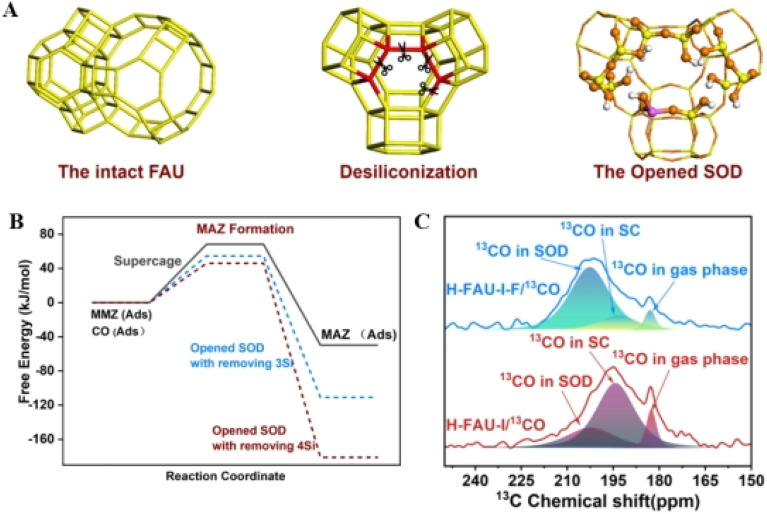
Theoretical calculation for the DMM adsorption and CO interaction reaction. (A) A model representing opened SOD constructed by removing Si atoms *via* mild NH_4_F treatment. (B) The free energy profile of the interaction of CO with methoxymethoxy groups for the formation of methoxyacetyl species in the supercage and opened SOD. (C) ^13^C MAS NMR spectra of ^13^CO adsorbed in H-FAU and H-FAU after NH_4_F treatment. Reproduced from ref. 85 with permission from American Chemical Society, copyright 2025.

In recent years, a series of extra-large-pore zeolites, *i.e.*, the ZEO family, have been developed,^91^ which provided the possibility to explore the effects of larger reaction space for DMM carbonylation. According to this, Gao *et al.*^27^ studied DMM carbonylation over ZEO-1, which possessed three-dimensional cross pores consisting of 16-MR and 12-MR channels, forming supercage structures with a maximum spherical diameter of 11.54 Å at three-dimensional channel intersections (Fig. 5). During the DMM carbonylation stability test, ZEO-1 exhibited much higher activity and stability, outperforming traditional FAU zeolites. The excellent performance of ZEO-1 is attributed to a lower carbonylation energy barrier and better diffusion properties due to extra-large pores and *in situ* synthesized high Si/Al ratios. Ni *et al.*^85^ also reported a strategy *via* mild NH_4_F treatment to open SOD cages of FAU, achieving optimal Brønsted acid microenvironments for DMM carbonylation (Fig. 6A). Over the modified FAU, 90% MMAc selectivity and a space-time yield of 0.7 g g^−1^ h^−1^ could be maintained over 500 h. The superior activity was attributed to enhanced acid strength, the enrichment of reactant CO and transition-state stabilization after the modification, as shown in Fig. 6B and C.

These results demonstrate that the DMM carbonylation reaction is efficient but quite sensitive to the local environment of zeolite containing supercages. For a better understanding of this reaction, further research should combine the influence of acid strength, acid density and their local environment in zeolite, and also more detailed analysis of the reaction kinetics.

Besides, there are some similarities and differences in DME and DMM carbonylation over zeolites. DME and DMM carbonylation both exhibit an obvious induction period because of the initial generation of reaction precursors.^21,85^ In the stable period, during the conversion of acyl intermediates to ester products through alkoxylation, regeneration of alkoxy precursors occurs at the same time, avoiding formation of methanol. However, for DME carbonylation, the formation of acetyl intermediates serves as the rate-determining step, whereas in DMM carbonylation, the rate-limited step is governed by zeolite topology. Specifically, in confined spaces such as the MFI framework, the conversion of acyl species is the rate-limited step, while in larger cavities like the supercages of FAU, the formation of acyl species determines the reaction rate. Despite their similar elementary steps, these two processes display substantially different activities. The reaction temperature for DME carbonylation could be 453–553 K, whereas DMM carbonylation occurs under much milder conditions (333–393 K). The superior activity observed in DMM carbonylation could probably be attributed to the enhanced stability of methoxyacetyl intermediates. A comprehensive comparative understanding of the mechanisms of DME and DMM carbonylation is suggested in the future for better understanding of zeolite-based ether carbonylation.

## 5. CO and CO_2_ coupling with alkanes to aromatics over H-zeolites

**Fig. 7:**
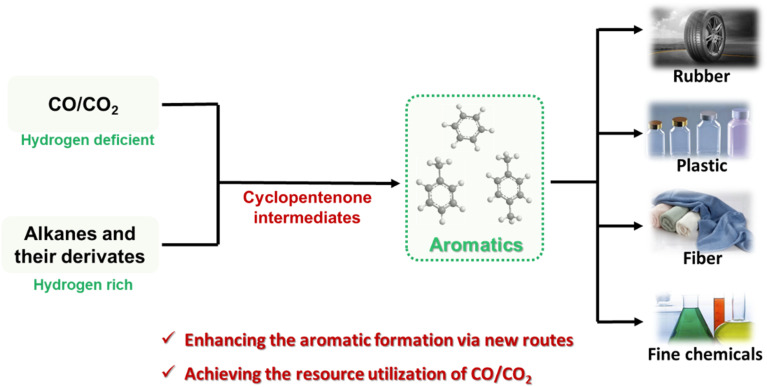
Schematic diagram of CO and CO_2_ coupling with alkanes or their derivatives to aromatics over H-zeolites.

Aromatic hydrocarbons such as benzene, toluene, and xylene (BTX) are widely used in the production of three major synthetic materials: synthetic fibers, synthetic resins, and synthetic rubber.^92^ In addition, they are also raw materials for solvents, dyes, insecticides, and adhesives.^93^ Aromatics are currently generated from naphtha catalytic reforming,^94,95^ limited by high operation costs and unsustainable feedstocks. Zeolite-based catalytic conversion of alkanes to aromatics shows promising performance and has attracted great interest in the past few decades. However, alkane reactants are characterized with a higher hydrogen to carbon ratio (>2) than that of aromatics, and therefore, based on the hydrogen-transfer reaction catalyzed by protons, the generation of hydrogen-deficient aromatics from alkanes will be accompanied by the generation of hydrogen-rich but low-valuable light alkanes.^96^ From the perspective of thermodynamics, the introduction of CO_*x*_ could shift the equilibrium of the alkane conversion towards the aromatic formation. G. Chen *et al.*^97^ conducted the thermodynamic analysis of ethane aromatization and ethane coupling conversion with CO_2_*via* HSC chemistry, and found that the addition of CO_2_ as a coreactant can help overcome the thermodynamic limitations of ethane conversion and shift the reaction equilibrium toward the aromatic products. Recently, it is interesting to find that co-feeding of CO and CO_2_ could inhibit the hydrogen-transfer reaction through nucleophilic attack to the alkyl cations. And cyclic ketenes will be generated as active intermediates for aromatics (Fig. 7). In this way, the small alkane generation could be suppressed and the carbon atoms of CO and CO_2_ are introduced into the aromatic rings.

### 5.1 The coupling reaction of CO with alkanes to aromatics over zeolites

In 2000, Luzgin and co-workers^37^ reported the reaction of alkanes such as propane and isobutane with CO to form carboxylic acids over zeolite ZSM-5 by *in situ* solid-state NMR spectroscopy. Wang *et al.*^99,100^ reported that carboxylic acids could be detected by *in situ* solid-state NMR spectroscopy during the co-reaction of methane/ethane with CO over Zn/ZSM-5. In these cases, the surface catalytic products of alkane carbonylation at low temperature were investigated through *in situ* spectroscopic investigation, demonstrating in theory the potential of zeolite in the realization of heterogeneous alkane carbonylation. However, the low efficiency of these carbonylation processes at low temperature severely hinders their further application.

**Fig. 8:**
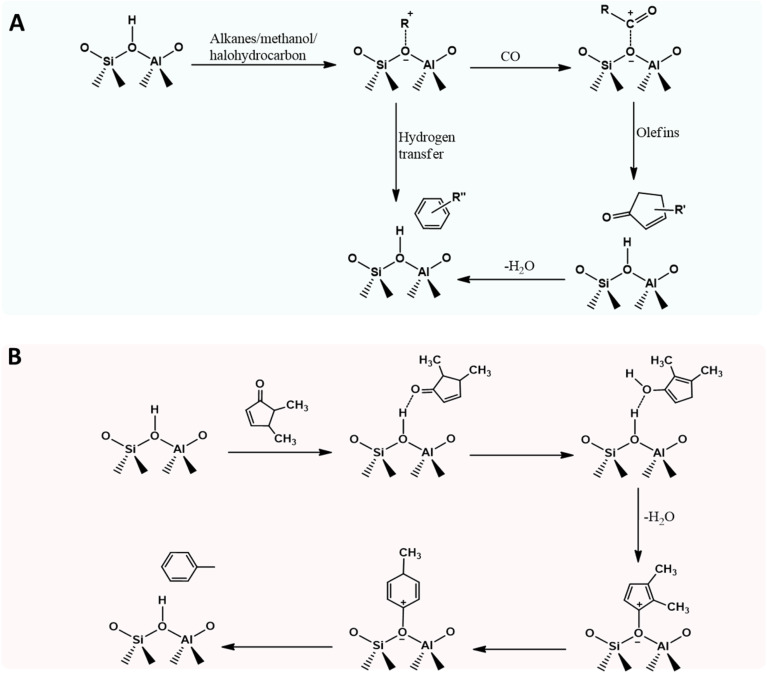
(A) Proposed mechanism of the coupling reaction of alkanes, methanol and halohydrocarbon with CO over acidic zeolites. (B) Proposed conversion pathways for the cyclopentenone intermediate into aromatics over acidic zeolite.^98^

Stepanov and coworkers^11^ reported that acylium cations generated from the CO insertion of carbonium ions could further react with olefins to produce unsaturated ketones over zeolite catalysts at ambient temperature. And Han *et al.* found that unsaturated ketones such as dimethyl cyclopentenone could be transformed into aromatics over zeolites.^98^ The proposed reaction pathway is listed in Fig. 8B; the adsorbed dimethyl cyclopentenone could isomerize to an enol, which could further dehydrate to produce dimethylcyclopentadienyl cations, accounting for the generation of aromatics *via* ring expansion and deprotonation.^101^ It is interesting to find that the cyclic ketene species could be converted to aromatics without loss of carbon atoms. These two chemical processes together indicate that alkane, olefin or alcohol substrates that can generate alkyl cations over zeolite catalysts might react with CO to produce aromatics *via* ketene intermediates.

Wei *et al.*^30^ first reported the coupling reaction of *n*-hexane with CO to form aromatics in a continuous flow reactor over ZSM-5. Compared to the conversion of *n*-hexane alone, the introduction of CO into *n*-hexane could significantly enhance the formation of aromatics and suppress the small alkane generation. An approximate 80% aromatics selectivity could be achieved under optimal conditions. *In situ* infrared spectroscopy and ^13^C isotope experiments revealed that CO could insert into carbonium ions to form acylium ions, which further reacted with olefins to form cyclopentenone intermediates, resulting in the aromatics formation (Fig. 8A). The C atoms from CO were ultimately incorporated into the aromatic products *via* a cyclopentenone intermediate, and O atoms of CO could eliminate the hydrogen of alkanes in the form of water (Fig. 8B), thereby regulating the H/C ratio of the products and promoting aromatics formation.

Subsequently, Wei *et al.*^96^ reported this coupling effect for promotion of aromatics formation was also revealed in systems of CO with other light alkanes (C_4_–C_6_). Besides, Wen *et al.* reported that methane could also be converted *via* coupling conversion with CO over Zn/ZSM-5 catalysts,^102^ and the high selectivity to aromatics (80%) could be achieved at 873 K, while 92% ethane selectivity could be obtained at a lower temperature of 673 K. After activation by zinc, CH_4_ was first converted into a methyl compound and then transferred to zeolite, where the carbonylation reaction occurred. The produced acetyl compound and/or acetic acid dehydrated to ketene, an intermediate for the formation of ethylene and aromatic.

In addition to alkanes, other substrates (methanol and halogenated alkanes) that can generate carbonium ions over zeolites could also undergo coupling reactions with CO to produce aromatics. Chen *et al.*^103^ investigated the coupling reaction of CO with methanol over ZSM-5 zeolites. An approximate 40% aromatics selectivity with a 53% C_2_–C_4_ paraffin selectivity was obtained during methanol conversion under a N_2_ atmosphere, and while methanol was co-fed with CO, high aromatics (∼80%) selectivity could be achieved over ZSM-5. Carbonyl-containing compounds such as acetic acid and MAc were generated from the reaction of CO with methanol, and these carbonylation intermediates could react with olefins to produce cyclopentenone species, serving as active intermediates for aromatic production. Moreover, the coupling reaction of CO with halogenated alkanes such as CH_3_Cl and C_2_H_5_Cl has also been proven to promote the formation of aromatics through similar reaction mechanisms,^36,104^ in which C atoms from CO were ultimately incorporated into the aromatics rings *via* cyclopentenone intermediates, leading to a dramatic drop in alkanes and a significant increase in aromatics selectivity.

In summary, the coupling reactions of alkanes, methanol and halogenated alkanes with CO over acidic zeolites provide a new route for aromatics formation (Fig. 8A), in which CO could react with carbonium ions to form acylium cations, and these acylium cations could further react with olefins to produce cyclopentenone species, leading to aromatics formation *via* dehydration and isomerization reactions. Compared with traditional aromatics formation *via* hydrogen transfer of olefins, the coupling route could reduce the formation of small alkanes and significantly enhance the aromatics selectivity, because the O atoms of CO could eliminate the hydrogen of olefins in the form of water, and the C atoms of CO could be incorporated into aromatics.

### 5.2 The coupling reaction of CO_2_ with alkanes to aromatics over zeolites

In addition to the promotion of aromatic formation by coupling alkanes with CO *via* oxygen-containing intermediates, the introduction of CO_2_ into the light alkane conversion to produce aromatics provides a novel and potential approach for direct utilization of CO_2_.

**Fig. 9:**
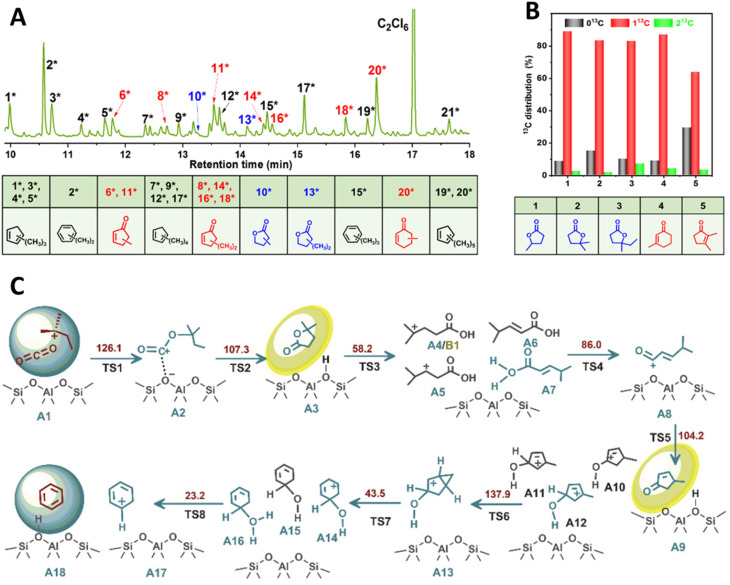
The coupling reaction of light alkanes (C_4_–C_6_) and CO_2_ over acidic ZSM-5. (A) GC-MS analysis of retained species occluded in spent ZSM-5. (B) ^13^C distribution of oxygenated compounds occluded in spent zeolite after the coupling reaction of *n*-butane with ^13^CO_2_. (C) Proposed mechanism of the aromatic formation for the coupling conversion of *n*-butane and CO_2_ over ZSM-5. Reproduced from ref. 31 with permission from Elsevier, copyright 2023.

The coupling reaction of light alkanes (C_4_–C_6_) and CO_2_ over acidic ZSM-5 zeolite was investigated.^31^ Compared with the conversion of light alkanes in He, the selectivity of aromatics is significantly increased for the CO_2_ coupling reactions. A CO_2_ conversion of 17.5% and *n*-butane conversion of 100% with an aromatic selectivity of 80% could be achieved under selected conditions. Methyl-substituted lactones (MLTOs) and methyl-substituted cycloalkenones (MCEOs) were generated from CO_2_ conversion by ^13^C isotope experiments (Fig. 9A and B) and were the key intermediates during the coupling reaction. Mechanistic experiments (Fig. 9C) unraveled that CO_2_ could be incorporated into MLTOs *via* the direct C–C bond-forming reactions, and could be further converted into MCEOs accounting for aromatics generation, and carbon atoms of CO_2_ could finally be incorporated into aromatic products. Moreover, the aromatization of propane,^32^ cyclohexane,^105^ and chloromethane^35^ could also be enhanced by coupling with CO_2_ over acidic zeolite, in which CO_2_ could enter the aromatic products through the formation of oxygenated intermediates, boosting the aromatics formation.

Due to the weak activation ability of CO_2_ over H-type zeolites, the introduction of metals such as Zn^106–108^ and Ga^33,109^ into zeolites could significantly boost CO_2_ conversion and enhance the aromatics selectivity. Feng *et al.*^33^ reported that an 80.76% aromatic selectivity at 99.57% cyclohexane conversion could be achieved over Ga-MFI during the CO_2_ and cyclohexane coupling reaction. ^13^C isotope experiments revealed that 56.4% of aromatic carbon originates from CO_2_. The Ga species were involved in reversible coordination switching between oxidized (Ga–O) and reduced (Ga–H) states, promoting CO_2_ and C–H activation. A summary of the recently reported literature studies about coupling reactions of alkanes with CO_2_ is shown in Table 1; compared with acidic zeolites, the metal-modified zeolites could significantly enhance CO_2_ conversion under mild conditions, incorporating more C atoms from CO_2_ into aromatic products and enhancing the generation of aromatics. Besides, CO_2_ could enhance the dehydrogenation of alkanes over metal-modified zeolites by consuming the released H_2_*via* the reverse water–gas shift reaction, promoting the aromatics formation.^97^

**Table 1:** Recently reported literature studies about coupling reactions of alkanes with CO_2_ over zeolites or modified zeolites

Reactants and catalysts	Active centers	Reaction conditions	CO_2_ conversion and aromatics selectivity	The portion of C atoms in CO_2_ incorporated into aromatics
*n*-Butane-CO_2_, ZSM-5	BAS	550 °C, 2.5 MPa, *n*-butane : CO_2_ : Ar = 40 : 19 : 1, WHSV_*n*-butane_ = 2.6 h^−1^	17.5% and 80%	10–15% (ref. 31)
Cyclohexane-CO_2_, Ga-MFI	GaH_*x*_ species & BAS	550 °C, 0.1 MPa, cyclohexane : CO_2_ : N_2_ = 1 : 8 : 2, WHSV = 1 h^−1^	7.4% and 80.76%	56.4% (ref. 33)
C_4_H_8_–CO_2_, ZnCrAlO_*x*_-ZSM-5	ZnCrAlO_*x*_ & BAS	500 °C, 1.0 MPa, *n*-butene : CO_2_ : Ar : N_2_ = 2 : 20 : 5 : 73, WHSV_butene_ = 0.4 h^−1^	10.5% and 80.0%	43.4% (ref. 108)
*n*-Butane-CO_2_, Zn/ZSM-5	[Zn–O–Zn]^2+^ & BAS	550 °C, 2.5 MPa, *n*-butane : CO_2_ : Ar = 19 : 20 : 1, WHSV_*n*-butane_ = 1.7 h^−1^	26.5% and 69.1%	13% (ref. 107)
Propane-CO_2_, Ga/M-Z5	BAS and Ga–O species	550 °C, 0.1 MPa, C_3_H_8_/CO_2_ = 1 : 1, WHSV = 5.24 h^−1^	CO_2_ conversion 54% and BTX selectivity 63%	Not mentioned^34^

The coupling reaction of CO_2_ with alkanes and their derivatives over acidic zeolites provides a novel pathway for aromatics formation *via* methyl-substituted lactones and methyl-substituted cycloalkenone intermediates. During the coupling reaction, CO_2_ can insert into the carbenium ions generated from alkane cracking to form alkyl carbonate species, which can further cyclize into methyl-substituted lactones. These lactones are key precursors for the formation of methyl-substituted cycloalkenone intermediates, resulting in the aromatics formation. On the whole, the O atoms from CO_2_ could consume H atoms from alkanes to form H_2_O, while the C atoms from CO_2_ could be incorporated into aromatics, thereby promoting the generation of aromatics and suppressing the formation of small alkanes.

## 6. Summary and future perspectives

The summary of CO_*x*_ coupling reactions over zeolites shows that the protons within confined zeolite channels or cages can catalyze: olefin/alcohol carbonylation to branched acids, olefin carbonylation to ketenes, DME carbonylation to MAc and acetone, methanol/CH_3_Cl carbonylation to acetic acid, DMM carbonylation to MMAc, and alkane coupling with CO_*x*_ to aromatics. For all these processes, the reaction begins with alkyl cation precursors, and the following formation of acyl intermediates play a key role in conversion of CO_*x*_. Transformation of acyls into final products of acids, ketenes, esters and aromatics requires nucleophilic attack from species such as water, olefins, ethers, *etc.* These cases show the great potential of zeolite catalysts in producing chemicals by direct conversion utilization of the attractive CO_*x*_ resources. However, the following aspects need to be considered in the future for a systematic understanding of the reaction mechanism and for better development of this field.

(1) ZSM-5 zeolite catalysts have been reported to catalyze the carbonylation of olefins/alcohols to acids, and tertiary acid products could be detected over the zeolite surface. Future research is suggested to explore whether zeolites can catalyze the carbonylation of olefins or alcohols to primary or secondary acidscompounds, and if so, how to control carbonylation activity and selectivity in this series of Koch-type carbonylation. In addition, the carbonylation processes of olefins, alcohols and alkanes have only been identified by *in situ* spectroscopic investigation, while the low efficiency of these carbonylation processes severely hinders their further application. Efforts need to be made for significant improvement to improve the efficiency of these carbonylation reactions for potential application in the future.

(2) There is a strict reaction space matching effect for DME carbonylation and all the carbonylation mediated with acetyl cations that occur selectively in 8-MR side pockets of MOR zeolite. While for other processes like olefin/alcohol carbonylation to acids, DMM carbonylation to MMAc and alkane coupling with CO_*x*_ to aromatics, it remains unclear whether there exists such a strict reaction space matching effect. And investigation into the formation and stabilization of different acyl species within zeolite channels or cages should be considered.

(3) DME and DMM carbonylation reactions show common features from the point of view of the catalytic cycle, and an induction period exists for both carbonylation processes. Understanding the induction reaction behavior and their relationships with the zeolitic confinement effect for both generation and conversion of acyl intermediates should be taken into account.

(4) Cyclic ketenes and lactones have been proposed as the key intermediates for coupling reactions of alkanes and CO_*x*_ to aromatics, through which C atoms from CO or CO_2_ were incorporated into aromatics products directly. However, the specific steps involved in the formation and conversion of cyclic ketenes or lactone intermediates still lack experimental and simulated evidence.

(5) Metal elements have been introduced to increase aromatics selectivity and CO_*x*_ conversion in the coupling reaction. The mechanism of metals in these reactions remains ambiguous. Which step was promoted by metals, the activation of alkanes, the formation or the conversion of cyclic ketenes and lactone intermediates, warrants further investigation. Besides, coupling alkanes with CO_2_ to produce aromatics can promote alkane activation by eliminating hydrogen *via* the reverse water-gas shift reaction, while the generated water may affect the catalytic stability of the zeolite catalyst. Therefore, improvement of the hydrothermal stability of zeolites should be considered in the future for these coupling reaction systems.

## Author contributions

C. C. Wei conducted the literature research, designed the figures, and wrote the manuscript. S. l. Gao was responsible for reviewing. L. Qi and Z. M. Liu conceived the perspective, supervised the project and revised the manuscript.

## Conflicts of interest

There are no conflicts to declare.

## Data Availability

No new data were created or analysed in this study. Data sharing is not applicable to this article.
